# Supplementary material for: The Prognostic Value of Eight Comorbidity Indices in Older Patients with Cancer: The ELCAPA Cohort Study
Source: Cancers (Basel). 2022 Apr 29;14(9):2236. doi: 10.3390/cancers14092236 (PMC9105640; doi:10.3390/cancers14092236)
Supplement: Supplementary file 1 [file cancers-14-02236-s001.zip › cancers-1687513-supplementary.pdf]

Table S1. Baseline Demographic, Functional, Oncological characteristics and comorbidities according to different scores.

		n=510	%	Scores weighs							NCI combined		
				CCI	Romano	CCI-Quan	CCI-Age	Elixhauser	CIRS-G	Gagne	Sein	Prostate	Colorectal
	Age												
	< 80	243	47,6				3						
	[80-90[	249	48,8				4						
	≥ 90	18	3,5				5						
**Cancer items**	Any tumor	510	100,0	2	2	2	2	4		1			
	Solid cancer	466	91,6										
	Lymphoma	43	8,4					9					
	Metastatic cancer	213	41,8	6	6	6	6	12		5			
**Conditions**	High blood pressure	333	65,3						HTA/Vascular 1-4	-1			
	Peripheral vascular disorder	138	27,1	1	1		1	2		1	0,22	0,36	0,28
	Myocardial infarction	99	19,4	1	1		1				0,40	0,05	-0,19
	Cardiac arythmias	148	29,0					5	Heart 1-4	1			
	Congestive heart failure	72	14,1	1	2	2	1	7		2	0,85	0,87	0,84
	Valvular disease	89	17,5					-1					
	Pulmonary circulation disorders	45	8,8					4	Respiratory 1-4	1			
	Chronic pulmonary disease	121	23,7	1	2	1	1	3		1	0,47	0,73	0,47
	Cerebrovascular disease	49	9,6	1	1		1		Neurological 1-4		0,71	0,27	0,55
	Neurodegenerative disorders	93	18,2					6					
	Hemiplegia	10	2,0	2	1	2	2			1			
	Paralysis	4	0,8					7			0,21	0,39	0,30
	Dementia	84	16,5	1	3	2	1			2	1,19	0,78	0,60
	Depression	164	32,2					-3	Psychiatry 1-4				
	Psychosis	4	0,8							1			
	Weight loss	299	58,6					6		2			
	Obesity	80	15,7					-4	Endocrine and metabolic 1-4				
	Uncomplicated diabete	67	13,1	1	1		1				0,45	0,24	0,44

Complicated diabete	51	10,0	2	2	1	2				1	0,02	0,44	0,30
Moderate to severe renal disease	285	55,9	2	3	1	2	5	Renal	1-4	2	1,19	0,68	0,97
Ulcer disease	32	6,3	1			1					0,23	-0,25	0,08
Liver disease	137	26,9	1	2	2	1	11	Liver	1-4	1			
Mild liver disease			3		4	3							
Blood loss anemias	106	20,8					-2						
Coagulopathy	71	13,9					3	Haematological	1-4	1			
Deficiency anemias	92	18,0					-2			1			
Fluid and electronic disorders	62	12,2					5			1			
Rheumatoid arthritis/ collagen vascular diseases,	22	4,3	1		1	1					0,75	0,09	-0,02
Alcohol abuse	19	3,7								1			
Drug abuse	0	0,0					-7						
ENT sphere									1-4				
Lower digestive									1-4				
Upper digestive									1-4				
Musculoskeletal									1-4				
Genitourinary									1-4				
HIV/ AIDS	1	0,2	6	4	4	6				-1			

ADL indicates Activity Daily Living; CCI indicates Charlson Comorbidity Index
